# Titania‐Coated Gold Nano‐Bipyramids for Blocking Autophagy Flux and Sensitizing Cancer Cells to Proteasome Inhibitor‐Induced Death

**DOI:** 10.1002/advs.201700585

**Published:** 2017-12-01

**Authors:** Hong‐Ye Wan, Jian‐Li Chen, Xingzhong Zhu, Liang Liu, Jianfang Wang, Xiao‐Ming Zhu

**Affiliations:** ^1^ State Key Laboratory of Quality Research in Chinese Medicine Macau Institute for Applied Research in Medicine and Health Macau University of Science and Technology Avenida Wai Long Taipa Macau SAR China; ^2^ Department of Physics The Chinese University of Hong Kong Shatin Hong Kong SAR China

**Keywords:** autophagy inhibition, bortezomib, cathepsin B, gold nano‐bipyramids, titanium dioxide

## Abstract

Targeting protein degradation is recognized as a valid approach to cancer therapy. The ubiquitin–proteasome system (UPS) and the autophagy–lysosome pathway are two major pathways for intracellular protein degradation. Proteasome inhibitors such as bortezomib are clinically approved for treating malignancies, but to date, they are still unsatisfactory for cancer therapy. This study identifies titania‐coated gold nano‐bipyramid (NBP/TiO_2_) nanostructures as an autophagic flux inhibitor, as the smallest NBP/TiO_2_ nanostructures induce significant autophagosome accumulation in human glioblastoma U‐87 MG cells via blocking the autophagosome–lysosome fusion process and inhibiting lysosomal degradation. Further study indicates that NBP/TiO_2_ nanostructures reduce the intracellular level of mature cathepsin B and directly inhibit the proteolytic activity of cathepsin B, thereby further inhibiting trypsin‐like proteolytic activity, which is a potential cotarget for UPS inhibition. NBP/TiO_2_ nanostructures interact synergistically with bortezomib to suppress the viability of U‐87 MG cells, as the combined treatment synergistically induces the intracellular accumulation of ubiquitinated protein and endoplasmic reticulum stress. In addition, photothermal therapy further synergistically reduces the cell viability. In summary, this study suggests that NBP/TiO_2_ nanostructures function as a promising anticancer agent in combination with proteasome inhibitors.

## 1 Introduction

The ubiquitin–proteasome system (UPS) and the autophagy–lysosome pathway are two major routes for intracellular protein degradation, which is strongly implicated in cancer pathogenesis and therapy. UPS degrades more than 80% of cellular proteins, especially short‐lived proteins. UPS‐mediated proteolysis consists of two steps: ubiquitination and proteasome‐mediated degradation.^1^ On the other hand, autophagy serves as the primary degradation route of long‐lived proteins, especially misfolded or aggregated proteins, and of damaged organelles. Intracellular proteins and organelles are engulfed in autophagosomes, which then fuse with lysosomes to form autolysosomes for degradation.^2^ Increasing evidence suggests that autophagy acts as a prosurvival mechanism in cancer cells under therapeutic stress, and it is associated with chemoresistance.^3^

Currently, targeting protein degradation has been recognized as a valid approach to cancer therapy. Bortezomib (Bor), a first‐generation proteasome inhibitor, which reversibly inhibits the chymotrypsin‐like activity of the β5‐subunit of the proteasome, has been approved for treating relapsed multiple myeloma.^4^ However, side effects (incidence >30%) and acquired resistance hamper its clinical applications. Furthermore, unfortunately, single proteasome inhibitor treatment has been proven unsatisfactory for solid tumors in clinical trials.^5^, ^6^ A recent clinical trial suggested that the anticancer effect of Bor is enhanced when it is combined with an autophagy inhibitor.^7^ To date, autophagy inhibitors including hydroxychloroquine (HCQ) and chloroquine (CQ) have been clinically viable. However, both HCQ and CQ can result in retinopathy,^8^ which may limit their clinical applications.

Nanomaterials hold great promise for cancer diagnosis and therapy. They can accumulate preferentially at tumor sites through targeting strategies.^9^ The endocytosis of nanomaterials most often culminates with lysosome internalization, so they may specifically affect the autophagy–lysosome pathway.^10^ There has been growing interest in nanomaterials for autophagy regulation. Quantum dots,^11^ carbon nanotubes,^12^ lanthanide oxide,^13^ cerium oxide,^14^ titanium dioxide (TiO_2_),^15^ and silver nanoparticles^16^ have been identified as autophagy inducers. However, nanomaterials for autophagy inhibition applications are still rare. Recently, citric acid‐capped gold,^17^ rare earth oxide,^18^ and iron oxide^19^ nanoparticles have been reported to induce autophagy dysfunction by blocking autophagy flux. However, in these studies, the mechanisms of these activities and their exact cellular targets are still unclear. The exploration of novel and effective autophagy inhibitors is highly desired.

The surface coatings and sizes of nanomaterials are believed to influence their effects on autophagy.^12^, ^13^, ^17^ Among various nanomaterials, gold nanostructures have the advantage of easy control of the surface coatings and particle sizes.^20^, ^21^ In this study, we synthesized gold nano‐bipyramids (NBPs) with different surface coatings and sizes and performed screening to identify autophagy inhibitors. In this study, we reported that TiO_2_‐coated NBP (NBP/TiO_2_) nanostructures act as a novel autophagy inhibitor in human glioblastoma U‐87 MG cells. Their inhibitory effect is highly dependent on the TiO_2_ surface coating and the particle size. NBP/TiO_2_ nanostructures block autophagosome–lysosome fusion and inhibit cellular proteolytic activity through the inhibition of cathepsin B (CTSB) maturation. Interestingly, they also inhibit trypsin‐like proteolytic activity, while Bor shows a poor inhibitory effect at this site of the proteasome. We found that the inhibition of autophagy flux by NBP/TiO_2_ nanostructures sensitizes cancer cells to Bor. In addition, gold nanostructures have been considered as candidate agents for cancer photothermal therapy.^20^, ^21^ Here, the synergistic anticancer effect of NBP/TiO_2_ nanostructure‐based photothermal therapy and Bor was investigated. Although TiO_2_ nanoparticles were previously reported to induce autophagy,^15^ autophagy flux blockade by TiO_2_‐coated gold nanostructures and the detailed mechanisms involved were confirmed in this study. To our knowledge, this study is the first to report that CTSB is the direct target protein for nanomaterial‐induced autophagy inhibition. The results from this study will aid in the rational design of nanomaterials for autophagy regulation, and they offer a novel strategy to enhance the therapeutic effect of proteasome inhibitors.

## 2 Results and Discussion

### 2.1 Screening Autophagy Inhibitors from Coated NBP Nanostructures

**Figure 1:**
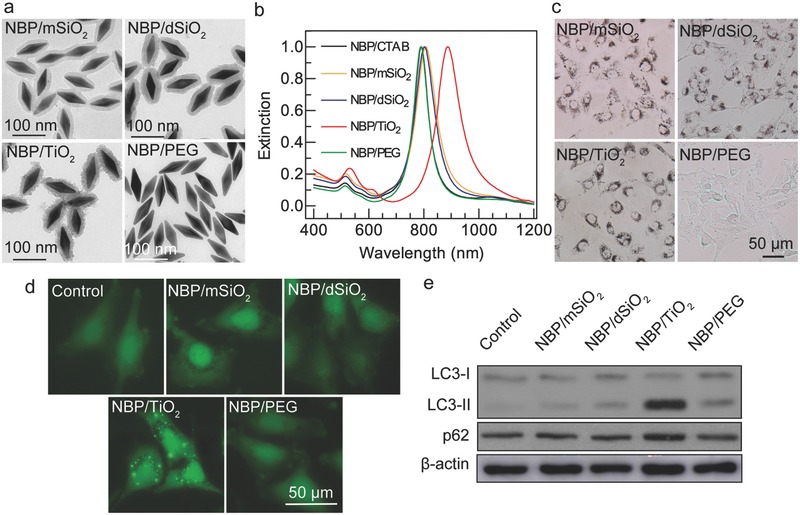
Screening and identification of NBP/TiO_2_ nanostructures as an autophagy inhibitor. a) TEM images of NBP/mSiO_2_, NBP/dSiO_2_, NBP/TiO_2_, and NBP/PEG nanostructure samples. b) Extinction spectra of the coated NBP samples in water. c) Optical images showing the internalization of the NBP samples in U‐87 MG cells after the cells were incubated with NBP samples with different coatings (60 µg Au mL^−1^) for 24 h. d) Accumulation of GFP‐LC3 puncta in U‐87 MG cells induced by NBP/TiO_2_ nanostructures. GFP‐LC3‐expressing U‐87 MG cells were treated with different NBP samples (60 µg Au mL^−1^) for 24 h. The distribution of GFP‐LC3 puncta was observed under a fluorescence microscope. e) NBP/TiO_2_ nanostructures increase intracellular LC3‐II and p62 protein levels. U‐87 MG cells treated with different NBP samples (60 µg Au mL^−1^, 24 h) were analyzed by western blotting for LC3 and p62 expressions.

NBPs capped with cetyltrimethylammonium bromide (CTAB) were synthesized and purified as described in a previous report.^22^ They were then coated with mesoporous silica (mSiO_2_), dense silica (dSiO_2_), TiO_2_, and poly(ethylene glycol) (PEG).^23^ The transmission electron microscopy (TEM) images and extinction spectra of all these NBP samples in aqueous solution are displayed in **Figure** **1**a,b. The NBP core has an average length and width of 87 ± 3 and 28 ± 1 nm, respectively. The longitudinal plasmon resonance wavelengths (LPRWs) are 790–886 nm.

During autophagy, intracellular contents are engulfed by double‐membrane vesicles named autophagosomes. The autophagosomes then fuse with lysosomes to form hybrid organelles named autolysosomes. The engulfed intracellular contents and the inner membrane of the autophagosomes are degraded inside the autolysosomes. Microtubule‐associated protein 1 light chain 3 (LC3) is an autophagosome marker. The conversion from LC3‐I to LC3‐II can be used to monitor autophagic activity, and the amount of LC3‐II positively correlates with the number of autophagosomes. We generated a U‐87 MG cell line constitutively expressing green fluorescent protein‐tagged LC3 (GFP‐LC3). In normal cells, GFP‐LC3 is distributed diffusely throughout the cytoplasm (Figure 1d). The redistribution of GFP‐LC3 from the cytosol to autophagosomes indicates the formation of autophagosomes, which are displayed as green fluorescent puncta.

Here, we performed a screen for autophagy modulators. First, the effects of the above synthesized NBPs with various surface coatings on autophagy were compared, as the surface chemistry of nanomaterials plays a vital role in their autophagy regulation function.^12^, ^13^ Among the four coated NBP samples, only the NBP/TiO_2_ nanostructure induced dramatic increases in GFP‐LC3 puncta (Figure 1d) and LC3‐II expression (Figure 1e).

Autophagosome accumulation can result from either autophagy activation by the upstream process or the blockade of autophagic flux at the later stage. As shown in Figure S1 (Supporting Information), accumulated autophagosomes (GFP‐LC3 puncta) can be induced by either the autophagy inducer rapamycin (Rap) or autophagy inhibitors such as bafilomycin A1 (BafA1) and CQ. To distinguish these two possibilities, we investigated the cellular level of p62, a ubiquitin‐binding protein that is delivered to lysosomes for degradation. An enhanced p62 protein level has been regarded as an indicator for the blockade of autophagic flux.^24^ Figure 1e shows that treatment with NBP/TiO_2_ nanostructures (60 µg Au mL^−1^, 24 h) induced a marked increase in the p62 level, reflecting an inhibition of autophagic flux. However, the other three NBP samples did not affect the p62 expression. Though the cellular uptake efficiencies for NBP/mSiO_2_ and NBP/TiO_2_ nanostructures were similar (Figure 1c), their effects on autophagy were different. These results suggest that NBP/TiO_2_ nanostructures are an autophagic flux inhibitor, and their effect is highly dependent on the surface TiO_2_ coating.

**Figure 2:**
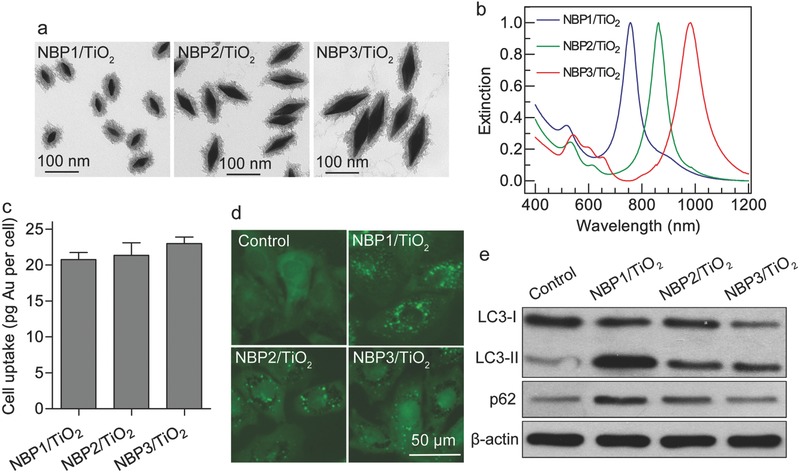
Size‐dependent autophagy inhibition by NBP/TiO_2_ nanostructures. a) TEM images of NBP1/TiO_2_, NBP2/TiO_2_, and NBP3/TiO_2_ nanostructures. The lengths of the NBP cores were 47 ± 4, 95 ± 5, and 142 ± 8 nm, respectively. b) Extinction spectra of the three NBP/TiO_2_ samples in water. c) Intracellular Au contents in U‐87 MG cells measured by inductively coupled plasma mass spectrometry (ICP‐MS). d) Size‐dependent accumulation of GFP‐LC3 puncta in U‐87 MG cells induced by NBP/TiO_2_ nanostructures. GFP‐LC3‐expressing U‐87 MG cells were treated with different NBP/TiO_2_ nanostructure samples (60 µg Au mL^−1^) for 24 h. The distribution of GFP‐LC3 puncta was observed under a fluorescence microscope. e) NBP1/TiO_2_ nanostructures induced the highest level of intracellular LC3‐II and p62. U‐87 MG cells treated with different NBP/TiO_2_ samples (60 µg Au mL^−1^, 24 h) were analyzed by western blotting for LC3 and p62 expressions.

Second, the effect of NBP/TiO_2_ nanostructures on the number of GFP‐LC3 puncta was revealed to depend on the particle size. Three NBP samples of different sizes were synthesized and purified and were named NBP1, NBP2, and NBP3. Their lengths were 47 ± 4, 95 ± 5, and 142 ± 8 nm, and their widths were 20 ± 2, 33 ± 2, and 42 ± 3 nm, respectively (Table S1 of the Supporting Information; **Figure** **2**a). After TiO_2_ coating, the LPRWs of the NBP1/TiO_2_, NBP2/TiO_2_, and NBP3/TiO_2_ nanostructures were 758, 862, and 954 nm, respectively (Figure 2b). Among these three NBP/TiO_2_ nanostructures, the smallest NBP1/TiO_2_ sample possessed the strongest autophagosome accumulation ability (Figure 2d) and induced much greater LC3‐II conversion and p62 accumulation in U‐87 MG cells (Figure 2e). A previous study suggested that gold nanoparticles induce autophagosome accumulation through size‐dependent nanoparticle uptake.^17^ However, in this study, the cellular uptake efficiencies of these three NBP/TiO_2_ samples were similar, as indicated by intracellular gold content measurement (Figure 2c). For the following studies, NBP1/TiO_2_ nanostructures were used.

### 2.2 NBP/TiO_2_ Nanostructures Block Autophagic Flux

**Figure 3:**
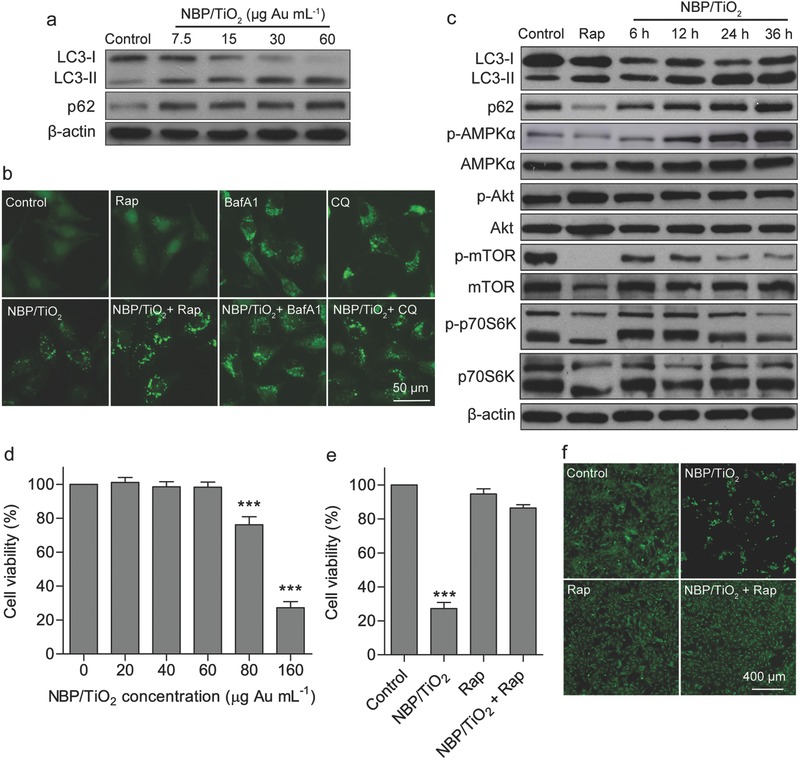
NBP/TiO_2_ nanostructures block autophagic flux. a) Concentration‐dependent autophagy inhibition by NBP/TiO_2_ nanostructures. U‐87 MG cells were incubated with NBP/TiO_2_ (0–60 µg Au mL^−1^) for 24 h, and then western blotting analysis was performed to detect LC3 and p62 expressions. b) LC3 turnover assay. GFP‐LC3‐expressing U‐87 MG cells were treated with NBP/TiO_2_ nanostructures (60 µg Au mL^−1^) in the presence or absence of Rap (1 × 10^−6^ m), BafA1 (10 × 10^−9^ m), or CQ (20 × 10^−6^ m) for 24 h, followed by fluorescence imaging. c) NBP/TiO_2_ nanostructures activate the AMPK/mTOR pathway. U‐87 MG cells were incubated with NBP/TiO_2_ (60 µg Au mL^−1^) for 6–36 h, and then western blotting analysis was performed to evaluate the expression of key proteins. d) Cell viability of U‐87 MG cells determined by 3‐(4,5‐dimethylthiazol‐2‐yl)‐2,5‐diphenyltetrazolium bromide (MTT) assay after incubation with NBP/TiO_2_ nanostructures (0–160 µg Au mL^−1^) for 72 h. Rap prevented the induction of cell death by NBP/TiO_2_ nanostructures. After U‐87 MG cells were treated with Rap (1 × 10^−6^ m) and/or NBP/TiO_2_ nanostructures (160 µg Au mL^−1^, 72 h), the cell viability was determined by e) MTT assay and f) calcein acetoxymethyl ester (calcein AM) staining. Live cells were stained with green fluorescence by calcein AM. The data shown represent the mean ± S.E.M., ***P < 0.001.

Variation in the dose of nanomaterials may lead to different autophagic effects. For example, pH‐sensitive polymeric nanoparticles induce autophagy at low concentration, while a high dose blocks autophagic flux.^25^ In our study, dose‐dependently (**Figure** **3**a) and time‐dependently (Figure 3c) increased LC3‐II level and p62 levels were observed after treatment with NBP/TiO_2_ nanostructures. Importantly, the lowest dose (7.5 µg Au mL^−1^) resulted in significant p62 accumulation (Figure 3a), revealing autophagy inhibition, and autophagy flux blockade occurred as early as 12 h after the treatment (Figure 3c).

To further clarify the effect of NBP/TiO_2_ nanostructures on autophagic flux, an LC3 turnover assay was carried out as described by Mizushima et al.^24^ If autophagy is inhibited, cotreatment with an autophagy inducer will increase the number of autophagosomes. The GFP‐LC3‐expressing U‐87 MG cells were treated with NBP/TiO_2_ nanostructures in the presence or absence of Rap, BafA1, or CQ. In NBP/TiO_2_ nanostructure‐treated cells, cotreatment with Rap further increased the number of GFP‐LC3 puncta (Figure 3b). By contrast, in the cells cotreated with BafA1 or CQ, the number of GFP‐LC3 puncta was not affected by the presence of NBP/TiO_2_ nanostructures (Figure 3b). In Rap‐treated cells, cotreatment with BafA1 or CQ significantly increased the number of GFP‐LC3 puncta (Figure S1, Supporting Information). These results therefore support the conclusion that NBP/TiO_2_ nanostructures block autophagic flux.

The blockade of autophagic flux decreases the recycling of cellular fuels, which eventually leads to reduced energy supply.^26^ To follow this phenomenon, we measured the intracellular production of adenosine triphosphate (ATP). The intracellular ATP level was significantly reduced in the cells treated with NBP/TiO_2_ nanostructures (30 or 60 µg Au mL^−1^) for 48 h (Figure S2, Supporting Information), but the NBP/TiO_2_ nanostructures at these two concentrations were not cytotoxic, as shown in Figure 3d. Adenosine monophosphate (AMP)‐activated serine/threonine protein kinase (AMPK) is a sensor of cellular energy status that is activated under low intracellular ATP conditions. NBP/TiO_2_ nanostructures caused a significant upregulation of AMPKα phosphorylation at residue T172 and a downregulation of mammalian target of rapamycin (mTOR) or p70S6K phosphorylation in a time‐dependent manner (Figure 3c). This result indicates that NBP/TiO_2_ nanostructures activate the AMPK/mTOR pathway, which is an important pathway involved in autophagy regulation. However, NBP/TiO_2_ nanostructures did not alter the Akt phosphorylation level at different time points (Figure 3c), indicating that they do not affect the PI3K (type I)/Akt/mTOR pathway.

Though the role of autophagy in cancer development is controversial,^27^ increasing evidence supports the idea that autophagy is a prosurvival mechanism by which cancer cells resist many cellular stresses such as starvation, hypoxia, and low pH.^3^, ^26^ It helps cells to remove damaged organelles and misfolded proteins and meanwhile provides substrates and energy for cancer cell survival. This view is supported by this study. NBP/TiO_2_ nanostructures did not affect the viability of U‐87 MG cells at 60 µg Au mL^−1^, but they significantly inhibited cell proliferation at concentrations above 80 µg Au mL^−1^ (Figure 3d). NBP1/TiO_2_ nanostructures at 160 µg Au mL^−1^ even induced more than 70% death. Interestingly, cotreatment with Rap (1 × 10^−6^ m) significantly restored the cell viability (Figure 3e,f). On the other hand, NBP/TiO_2_ nanostructures showed synergistic cytotoxicity with CQ (Figure S3, Supporting Information). These results indicate that NBP/TiO_2_ nanostructure‐induced cytotoxicity can be attributed to the autophagy inhibitory effect. Taken together, these results provide strong evidence that NBP/TiO_2_ nanostructures act as a potent autophagy inhibitor.

### 2.3 NBP/TiO_2_ Nanostructures Inhibit Autophagosome–Lysosome Fusion

**Figure 4:**
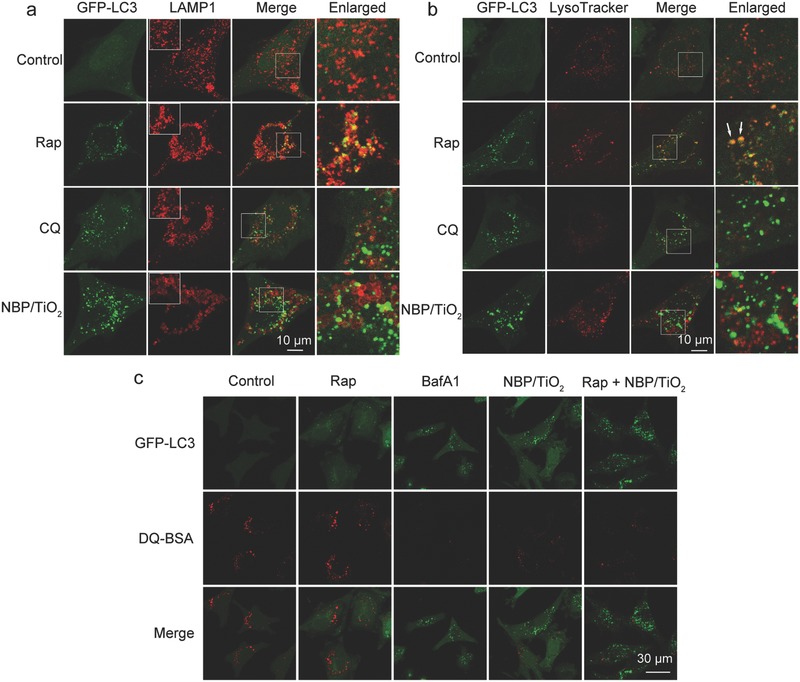
NBP/TiO_2_ nanostructures inhibit autophagosome–lysosome fusion and lysosomal proteolytic activity. a) Immunofluorescent staining with anti‐LAMP1 and b) live cell LysoTracker Red imaging of GFP‐LC3‐expressing U‐87 MG cells treated with NBP/TiO_2_ nanostructures. Autophagosomes and lysosomes failed to fuse in NBP/TiO_2_ sample treated cells. U‐87 MG cells stably expressing GFP‐LC3 were treated with NBP/TiO_2_ nanostructures (60 µg Au mL^−1^), Rap (1 × 10^−6^ m) for 24 h, or CQ (50 × 10^−6^ m) for 4 h, followed by fluorescent imaging under a confocal microscope. Fusion between autophagosomes and lysosomes is clearly shown in yellow (indicated by white arrows) in the Rap‐treated cells, but a nearly complete separation between autophagosomes and lysosomes can be observed in CQ‐ or NBP/TiO_2_ nanostructure‐treated cells. The dilated lysosomes are evident in the NBP/TiO_2_ nanostructure‐treated cells. c) NBP/TiO_2_ nanostructures inhibit lysosomal proteolytic activity. U‐87 MG cells expressing GFP‐LC3 were pretreated with DQ‐BSA (10 µg mL^−1^) for 12 h. The cells were then washed with PBS and treated with NBP/TiO_2_ nanostructures (60 µg Au mL^−1^), Rap (1 × 10^−6^ m), or BafA1 (10 × 10^−9^ m) for 24 h. The cells were observed under a confocal microscope.

The final stage of autophagy is the fusion of autophagosomes with lysosomes. This step was investigated by staining U‐87 MG cells expressing GFP‐LC3 with LAMP1 (a marker for endosomal and lysosomal membranes) or LysoTracker Red (a dye specific for lysosomes). As a positive autophagy induction control, the GFP‐LC3 puncta induced by Rap were well colocalized with anti‐LAMP1 (**Figure** **4**a) or LysoTracker Red (Figure 4b), indicating fusion between autophagosomes and lysosomes. By contrast, the GFP‐LC3 puncta and lysosomal signals did not overlap in cells treated with CQ. Similar to CQ, NBP/TiO_2_ nanostructures blocked autophagosome–lysosome fusion (Figure 4a,b).

A low pH in the lysosome is required for the activity of lysosomal enzymes. The fluorescence intensity of LysoTracker Red positively correlates with the acidity of lysosomes. CQ acts as a potent lysosomal deacidification agent and thus induced a significant increase in the lysosomal pH value (Figure 4b). Unlike CQ, NBP/TiO_2_ nanostructures did not alter the lysosomal acidity (Figure 4b), but they did induce clear lysosome dilatation (Figure 4a). Furthermore, the intracellular cytoskeleton component F‐actin is related to the fusion between autophagosomes and lysosomes.^28^ Similar to BafA1, NBP/TiO_2_ nanostructures disrupted the distribution of intracellular F‐actin (Figure S4, Supporting Information). These results suggest that the target intracellular organelle for NBP/TiO_2_ nanostructures is the lysosome.

Though some reports^17^, ^18^, ^19^ have suggested that the nanomaterial‐induced autophagosome accumulation occurs via autophagic flux blockade, the mechanisms have not been identified yet. In this study, we systematically studied the effect of NBP/TiO_2_ nanostructures on the lysosomal proteolytic activity, as the efficiency of lysosomal degradation determines the autophagic flux. The lysosomal proteolytic capacity was visualized with derivative‐quenched bovine serum albumin (DQ‐BSA), which is a self‐quenched lysosome degradation indicator. Proteolysis of this compound results in dequenching and the release of bright red fluorescent fragments. As shown in Figure 4c, dequenching of DQ‐BSA occurred in the control and especially in the Rap‐treated cells. By contrast, no dequenching of DQ‐BSA occurred in the cells treated with BafA1 or NBP/TiO_2_ nanostructures. This result suggests that NBP/TiO_2_ nanostructures inhibit the basal level of lysosomal proteolytic activity.

### 2.4 NBP/TiO_2_ Nanostructures Inhibit CTSB Activity

**Figure 5:**
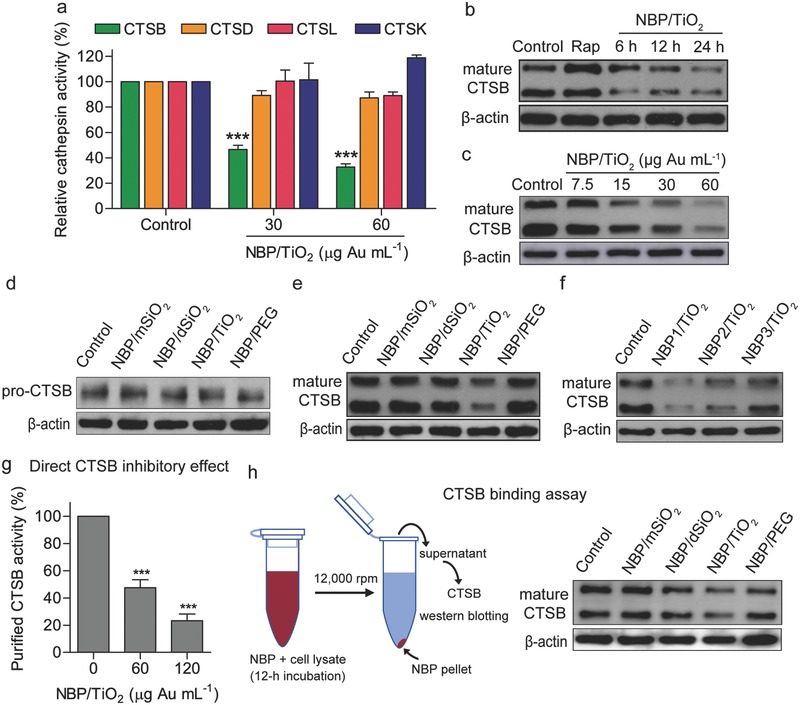
NBP/TiO_2_ nanostructures inhibit CTSB activity. a) Effect of NBP/TiO_2_ nanostructures on cathepsin activities. U‐87 MG cells were treated with NBP/TiO_2_ nanostructures (60 µg Au mL^−1^) for 24 h, and the activities of cathepsin B, D, L, and K were determined using specific fluorescent substrates. b) Time‐dependent and c) concentration‐dependent mature CTSB protein reduction by NBP/TiO_2_ nanostructures. U‐87 MG cells were incubated with NBP/TiO_2_ nanostructures (60 µg Au mL^−1^ for 6–24 h, or 0–60 µg Au mL^−1^ for 24 h), and western blotting analysis was performed to detect mature CTSB expression. d) NBP samples do not affect the protein level of pro‐CTSB. The reduction of mature CTSB by NBP/TiO_2_ nanostructures was dependent on the e) TiO_2_ coating and f) size, as the smallest NBP1/TiO_2_ nanostructures led to the lowest intracellular CTSB level. CTSB expression was detected by western blotting analysis after cells were incubated with coated NBPs (60 µg Au mL^−1^) for 24 h. g) NBP/TiO_2_ nanostructures inhibit the activity of purified human CTSB. Purified human CTSB (0.1 µg) was pretreated with NBP/TiO_2_ nanostructures (60 or 120 µg Au mL^−1^) for 12 h, followed by incubation with Z‐RR‐AMC (25 × 10^−6^ m) for 30 min, and the CTSB activity was determined by detecting the fluorescent intensity of the solution. h) NBP/TiO_2_ nanostructures bind to mature CTSB in the cell lysate. The cell lysate of U‐87 MG cells (2.7 µg protein µL^−1^, 70 µL) was incubated with NBPs with different coatings (20 µg Au) for 12 h at 4 °C. The supernatant was collected after centrifugation (12 000 rpm, 10 min), and the mature CTSB level was determined by western blotting analysis. The data shown represent the mean ± S.E.M., ***P < 0.001.

Cathepsins are the main lysosomal proteases required for the autophagic degradation process,^29^ and they are important for the death, proliferation, and invasion of human cancer cells.^30^ CTSB, cathepsin D (CTSD), and cathepsin L (CTSL) are the most abundant lysosomal proteases and participate directly in the execution of autophagy.^31^, ^32^, ^33^ A lack of CTSB results in an increase in the number and size of lysosomes and autophagosomes.^34^ Here, we investigated the effect of NBP/TiO_2_ nanostructures on these three cathepsins as well as on cathepsin K (CTSK), which is overexpressed in U‐87 MG cells.^35^ In vitro cathepsin assays showed that NBP/TiO_2_ nanostructures dramatically and selectively reduced the activity of CTSB (**Figure** **5**a). In addition, they even inhibited the Rap‐stimulated CTSB activity (Figure S5, Supporting Information). However, they did not inhibit the activities of the other three cathepsins.

CTSB is produced from a larger inactive precursor form, pro‐cathepsin B (pro‐CTSB). The activation of pro‐CTSB by conversion to mature CTSB occurs in the lysosome. The precursor is converted into a two‐chain form, resulting in subunits of 27 kDa, 24 kDa (heavy chain), and 5 kDa (light chain).^36^ Western blotting analysis (Figure 5b,c) indicated that NBP/TiO_2_ nanostructures dose‐dependently and time‐dependently reduced the level of intracellular mature CTSB protein (27 and 24 kDa). Even as little as 15 µg Au mL^−1^ of NBP/TiO_2_ nanostructures significantly reduced the production of mature CTSB (Figure 5c). The pro‐CTSB signal in the western blotting analysis results was quite weak, but Figure 5d shows that the NBP/TiO_2_ nanostructures did not affect the protein expression of pro‐CTSB. Furthermore, real time‐PCR analysis indicated that the NBP/TiO_2_ nanostructures did not alter the mRNA expression of pro‐CTSB (Figure S6, Supporting Information). The reduction in mature CTSB by the NBP/TiO_2_ nanostructures was dependent on the TiO_2_ coating, as the mSiO_2_‐, dSiO_2_‐ and PEG‐coated NBPs did not affect the intracellular level of mature CTSB (Figure 5e). Furthermore, the lowest level of mature CTSB was found for the cells treated with the smallest NBP1/TiO_2_ sample (Figure 5f).

To clarify whether NBP/TiO_2_ nanostructures have a direct effect on the proteolytic activity of CTSB, we performed an in vitro CTSB activity assay using purified human CTSB without centrifugation steps. We found that high concentrations of NBP/TiO_2_ nanostructures (≥60 µg Au mL^−1^) also directly inhibited the activity of CTSB (Figure 5g). We then incubated the lysate of U‐87 MG cells with NBPs with different coatings for 12 h, followed by centrifugation and western blotting analysis to determine the CTSB level in the supernatant. We found that the CTSB content in the cell lysate incubated with NBP/TiO_2_ nanostructures was reduced (Figure 5h), suggesting that NBP/TiO_2_ nanostructures can bind to mature CTSB.

Taken together, the above results suggest that NBP/TiO_2_ nanostructures are able to inhibit CTSB maturation even at low concentrations and can also bind to CTSB, resulting in the direct inhibition of CTSB activity at high concentrations.

### 2.5 NBP/TiO_2_ Nanostructures Inhibit Cellular Proteolytic Activity

UPS and autophagy were initially believed to be two independent systems associated with different degradation mechanisms, and challenging the proteolytic capacity of either system results in intracellular protein accumulation. Recently, cross‐talk between these two systems has emerged. For example, ubiquitin modification serves as a signal in both the proteasome and lysosome protein degradation pathways.^37^ Ubiquitinated proteins can be degraded by autophagy through p62 docking protein,^38^ and the autophagic protein LC3 can be processed by the 20S proteasome.^39^ Here, the effect of NBP/TiO_2_ nanostructures on UPS was studied.

**Figure 6:**
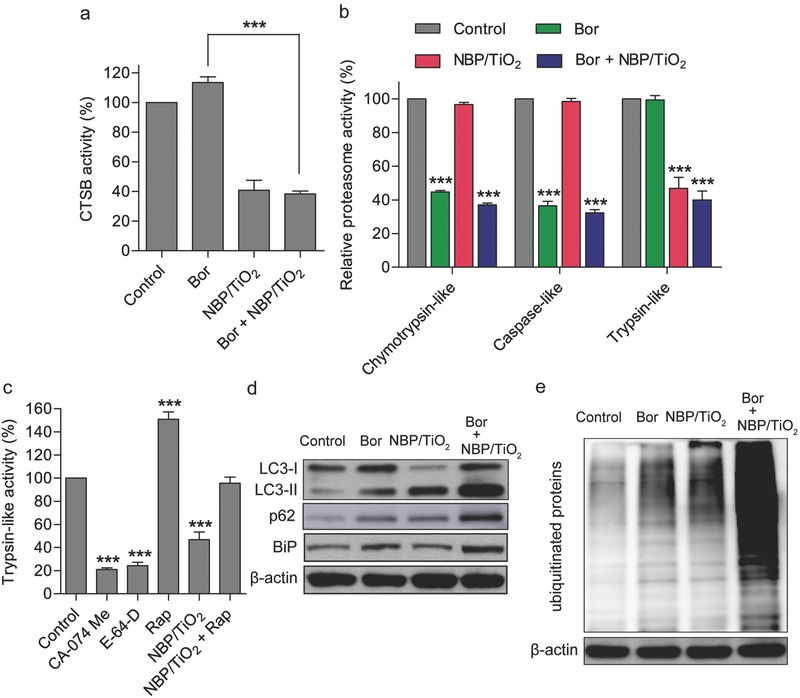
NBP/TiO_2_ nanostructures synergistically inhibit cellular proteolytic activity in combination with Bor. a) Effect of treatment with NBP/TiO_2_ nanostructures and/or Bor on CTSB activity. b,c) NBP/TiO_2_ sample and CTSB inhibitors inhibit trypsin‐like proteasome activity. After U‐87 MG cells were treated with NBP/TiO_2_ nanostructures (60 µg Au mL^−1^), Bor (10 × 10^−9^ m), Bor plus NBP/TiO_2_, CA‐074 Me (10 × 10^−6^ m), E‐64‐D (20 × 10^−6^ m), Rap (1 × 10^−6^ m), or Rap plus NBP/TiO_2_ for 24 h, the CTSB and proteasome activities of the cell lysates were determined by fluorogenic assays. d,e) The combination of NBP/TiO_2_ nanostructures and Bor synergistically inhibits intracellular protein degradation. After U‐87 MG cells were treated with NBP/TiO_2_ nanostructures (60 µg Au mL^−1^), Bor (10 × 10^−9^ m), and Bor plus NBP/TiO_2_ for 24 h, western blotting analysis was performed to evaluate the expression of LC3, p62, BiP, and ubiquitinated proteins. The data shown represent the mean ± S.E.M., ***P < 0.001.

The proteasome is a 26S enzyme complex consisting of a 20S core complex and a 19S regulatory complex. The 20S proteasome core contains three different types of active sites, including chymotrypsin‐like, trypsin‐like, and caspase‐like sites, which are located in three distinct units, β5, β2, and β1, respectively. Each subunit preferentially cleaves after hydrophobic (β5), basic (β2), or acidic (β1) residues in proteins to yield short peptides.^40^ Among the three proteolytic activities of proteasomes, the chymotrypsin‐like activity is rate‐limiting in protein breakdown by proteasomes, and thus, proteasome inhibitors including Bor and carfilzomib that target chymotrypsin‐like sites have been developed. As expected, Bor (10 × 10^−9^ m) significantly inhibits the intracellular chymotrypsin‐like and caspase‐like activities of proteasomes but does not affect the trypsin‐like activity (**Figure** **6**b). Interestingly, NBP/TiO_2_ nanostructures (60 µg Au mL^−1^) significantly inhibit the trypsin‐like activity, while they do not affect the chymotrypsin‐like and caspase‐like activities (Figure 6b). It has been reported that CTSB is responsible for trypsin activation in cells.^41^ CA‐074 Me and E‐64‐D are two common CTSB inhibitors, and we found that both of them markedly inhibit cellular trypsin‐like proteolytic activity (Figure 6c). Furthermore, Rap is able to restore trypsin‐like activity (Figure 6c). These results indicate that the inhibition of cellular trypsin‐like activity by NBP/TiO_2_ nanostructures can be attributed to their inhibitory effect against CTSB.

We hypothesized that the autophagy flux blockade by NBP/TiO_2_ nanostructures further induces toxic protein aggregation if combined with proteasome inhibitors. The synergistic effect of NBP/TiO_2_ nanostructures with the proteasome inhibitor Bor was studied. The combination of Bor and NBP/TiO_2_ nanostructures inhibits all three proteasomal and CTSB activities (Figure 6a,b), leading to the accumulation of p62 (Figure 6d). More importantly, NBP/TiO_2_ nanostructures show a superior synergistic effect with Bor, inducing ubiquitinated protein accumulation (Figure 6e). The possible mechanism is that the excess p62 accumulation induced by autophagy inhibition delays the delivery of ubiquitinated proteins to the proteasome, thereby inhibiting their clearance for proteasomal degradation.^42^ The accumulation of ubiquitinated proteins perturbs cellular homeostasis and induces cell death through endoplasmic reticulum (ER) stress.^43^ The binding immunoglobulin protein (BiP) is an essential regulator of ER homeostasis, and the expression of BiP is widely used as a marker of ER stress.^44^ As shown in Figure 6d, enhanced expression of BiP was observed in the cells treated with the combination of Bor and NBP/TiO_2_ nanostructures, indicating the activation of ER stress.

### 2.6 NBP/TiO_2_ Nanostructures Potentiate Bor‐Induced Cell Death

**Figure 7:**
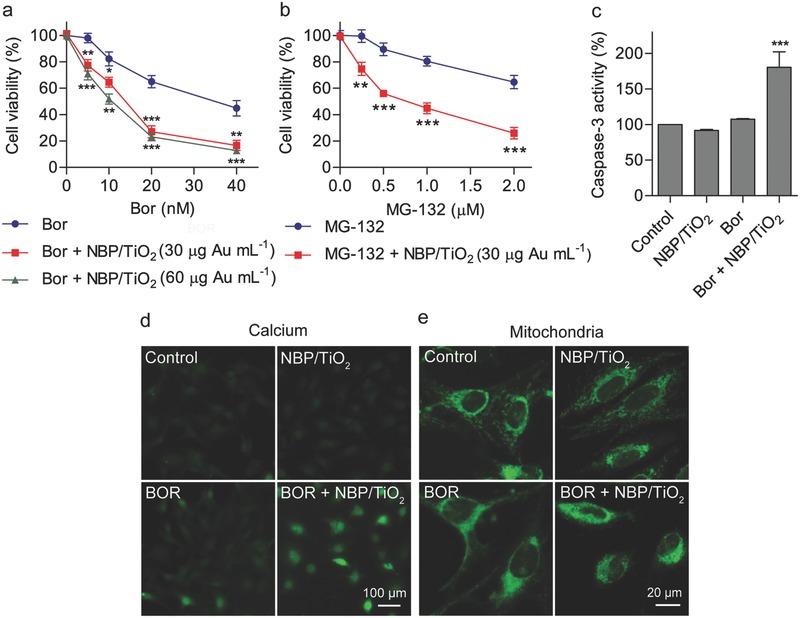
NBP/TiO_2_ nanostructures synergistically enhance the cytotoxicity of Bor. a,b) NBP/TiO_2_ nanostructures interact synergistically with proteasome inhibitor Bor or MG‐132 to suppress proliferation of U‐87 MG cells. U‐87 MG cells were treated with various concentrations of Bor (0–40 × 10^−9^ m) or MG‐132 (0–2 × 10^−6^ m) in the presence or absence of NBP/TiO_2_ nanostructures (30 or 60 µg Au mL^−1^) for 48 h, and the MTT assay was performed to evaluate cell viability. c) Caspase‐3 activation by Bor and NBP/TiO_2_ nanostructure combination treatment. U‐87 MG cells were treated with Bor (10 × 10^−9^ m) and/or NBP/TiO_2_ nanostructures (60 µg Au mL^−1^) for 24 h, and the caspase‐3 activity of the cell lysate was determined by a fluorogenic assay. Fluorescent imaging of d) intracellular calcium and e) mitochondria in the cells treated with Bor (10 × 10^−9^ m) and/or NBP/TiO_2_ nanostructures (30 µg Au mL^−1^). The data shown represent the mean ± S.E.M., *P < 0.05, **P < 0.01, and ***P < 0.001.

The simultaneous targeting of both arms of protein degradation represents a promising method for cancer therapy.^7^ We then tested whether the concomitant inhibition of autophagy by NBP/TiO_2_ nanostructures at a sub‐cytotoxic concentration can potentiate the anticancer effect of Bor in U‐87 MG cells. Here, NBP/TiO_2_ nanostructures (30 or 60 µg mL^−1^) interacted synergistically with Bor (5–40 × 10^−9^ m) to suppress the proliferation of U‐87 MG cells (**Figure** **7**a). Cotreatment with Bor and NBP/TiO_2_ nanostructures, specifically at doses of 10 × 10^−9^ m and 60 µg Au mL^−1^, respectively, resulted in reduced cell viability (51.8 ± 3.8)% compared to Bor alone ((82.2 ± 5.2)%, *P* < 0.01, Figure 7a), supporting the idea that NBP/TiO_2_ nanostructures sensitize U‐87 MG cells to Bor. NBP/TiO_2_ nanostructures (30 µg Au mL^−1^) also enhance the cytotoxicity of MG‐132, an earlier generation of proteasome inhibitor (Figure 7b). Prolonged ER stress results in the activation of apoptotic signaling.^45^ During ER stress, calcium (Ca^2+^) efflux from the ER increases the cytosolic Ca^2+^ level and disturbs the mitochondrial function.^46^ The cytochrome c released from the mitochondria forms an apoptosome complex with Apaf1 and caspase 9. This complex further activates the executioners caspase‐3 and caspase‐7, leading to apoptosis.^47^ Neither NBP/TiO_2_ nanostructures (60 µg Au mL^−1^) nor Bor (10 × 10^−9^ m) activated caspase‐3 activity after 24 h of treatment; however, combined treatment with Bor and NBP/TiO_2_ nanostructures significantly activated caspase‐3 activity (Figure 7c). The fluorescent imaging data showed that the combined treatment also induced a high level of intracellular Ca^2+^ (Figure 7d) and enhanced mitochondria injury (Figure 7e). The mitochondria show elongated filamentous structures in control or NBP/TiO_2_ nanostructure‐treated cells. Bor causes mitochondria to undergo fission and swelling. When it was combined with NBP/TiO_2_ nanostructures, even more severe aggregation of mitochondria was observed (Figure 7e). In addition, CA‐074 Me shows a similar synergistic anticancer effect with Bor (Figure S7, Supporting Information), indicating that Bor sensitization by NBP/TiO_2_ nanostructures can be assumed to be related to CTSB inhibition.

There remains a strong continued interest in improving the therapeutic effect of current proteasome inhibitors for treating myeloma and possible solid tumor cancers. Recently, proteasome inhibitors targeting the β1 (caspase‐like)^48^ or β2 (trypsin‐like) subunit^49^ were reported to sensitize malignant cells to Bor and carfilzomib and thus are considered as cotargets for UPS inhibition. In addition, in Bor‐resistant myeloma cells, the β2 proteasomal activity was upregulated compared with that in the nonresistant cells, indicating that inhibition of the proteasome β2 subunit could be of therapeutic value.^50^ However, the inhibition of the β2 proteasome subunit for therapeutic purposes has not been systemically explored, partly because cell‐permeable β2‐selective proteasome inhibitors are not available. In this study, NBP/TiO_2_ nanostructures, which selectively inhibit the trypsin‐like proteolytic activity, were first reported to possess a superior synergistic anticancer effect with Bor.

### 2.7 Photothermal Therapy‐Enhanced Cytotoxicity of Bor

**Figure 8:**
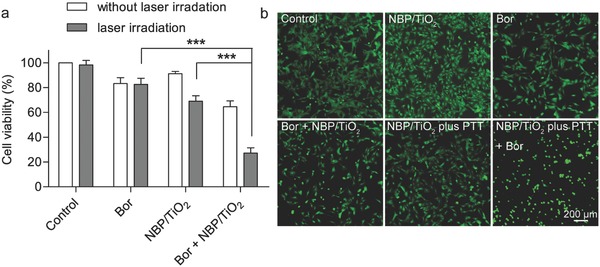
NBP/TiO_2_ nanostructure‐based photothermal therapy shows a synergistic anticancer effect with Bor. U‐87 MG cells were treated with photothermal therapy (808 nm laser irradiation, 3.7 W cm^−2^ for 2 min) with or without Bor (10 × 10^−9^ m, 48 h). Cell viability was determined by a) MTT assay and b) calcein AM staining. Live cells are stained with green fluorescence. The cells treated with photothermal therapy together with Bor show a clearly rounded morphology. The data shown represent the mean ± S.E.M., ***P < 0.001.

Clinical hyperthermia therapy has been used in combination with chemotherapy.^51^ It is performed with a moderate temperature elevation, and it can significantly enhance the cytotoxicity of chemotherapeutic agents.^52^ Previous studies also reported that hyperthermia therapy enhances the cytotoxicity of Bor.^53^, ^54^, ^55^ Gold nanostructure‐based photothermal therapy has recently attracted substantial attention for its selective and noninvasive nature. Photothermal therapy is induced by a near‐infrared (NIR) light laser, as NIR light with a wavelength longer than 650 nm is capable of deep tissue penetration.^56^ In this regard, we examined the efficacy of NBP/TiO_2_ nanostructure‐mediated photothermal therapy in combination with Bor. A low irradiation dose (3.7 W cm^−2^ for 2 min) was used in this experiment. We found the cell viabilities after monotherapy with photothermal therapy (3.7 W cm^‒2^ for 2 min) and Bor (10 × 10^−9^ m, 24 h) alone to be (69.0 ± 4.4)% and (83.3 ± 4.8)%, respectively. However, a combination of these two treatments synergistically reduced the cell viability to (27.3 ± 4.2)% (**Figure** **8**a). Nearly all of the cells were rounded after the combined treatment (Figure 8b). This result indicates that the simultaneous application of NBP/TiO_2_ nanostructure‐based photothermal therapy further synergistically enhances the anticancer effect of Bor. This combined treatment provides the possibility of reducing the dosage of proteasome inhibitors and even achieving enhanced anticancer effects. We believe that NBP/TiO_2_ nanostructures will offer great potential in cancer therapy.

## 3 Conclusions

In summary, we have identified NBP/TiO_2_ nanostructures as an autophagy flux inhibitor for sensitizing cancer cells to bortezomib. The autophagy inhibitory effect of NBP/TiO_2_ nanostructures is highly dependent on the TiO_2_ surface coating and the particle size. The target intracellular organelle for the NBP/TiO_2_ nanostructures is the lysosome, and they induce significant autophagosome accumulation in human glioblastoma U‐87 MG cells via blocking the autophagosome–lysosome fusion process. NBP/TiO_2_ nanostructures show a dramatic inhibitory effect against CTSB activity. They inhibit the maturation of CTSB and directly inhibit the proteolytic activity of CTSB by binding to mature CTSB. More importantly, NBP/TiO_2_ nanostructures also inhibit trypsin‐like proteolytic activity, and they synergistically enhance the anticancer effect of bortezomib. In addition, NBP/TiO_2_ nanostructure‐based photothermal therapy further enhances the anticancer effect. We believe that the combination of NBP/TiO_2_ nanostructures with bortezomib‐based chemotherapy will be useful for improving its efficacy.

## 4 Experimental Section

*Cell Culture*: U‐87 MG cells were cultured in alpha‐modified minimum essential medium (α‐MEM) containing 10% fetal bovine serum, 100 U mL^−1^ penicillin, and 100 µg mL^−1^ streptomycin at 37 °C in a humidified 5% CO_2_ atmosphere. GFP‐LC3 plasmid was introduced into U‐87 MG cells using the transfection reagent Hilymax, and a stable cell line was established and maintained in medium containing geneticin (500 µg mL^−1^).

*Preparation of CTAB‐Capped Au NBPs*: Au NBP samples were prepared using a seed‐mediated growth method.^22^ Briefly, the seed solution was made by adding freshly prepared, ice‐cold NaBH_4_ solution (0.01 m, 0.15 mL) into a mixture of HAuCl_4_ (0.01 m, 0.125 mL), trisodium citrate (0.01 m, 0.25 mL), and water (9.625 mL). The resultant seed solution was kept at room temperature for 2 h before use. The CTAB growth solution was prepared by the sequential addition of HAuCl_4_ (0.01 m, 2 mL), AgNO_3_ (0.01 m, 0.4 mL), HCl (1.0 m, 0.8 mL), and ascorbic acid (0.1 m, 0.32 mL) to aqueous CTAB solution (0.1 m, 40 mL). After gentle inversion stirring for 10 s, the seed solution was rapidly added to the growth solution. The resultant solution was mixed by stirring for 30 s and then kept at 30 °C overnight.

*Purification of CTAB‐Capped Au NBPs*: The Au NBP sample was centrifuged at 5000–9000 rpm for 10 min. The resultant precipitate was redispersed in aqueous cetyltrimethylammonium chloride solution (0.08 m, 30 mL), followed by the addition of AgNO_3_ (0.01 m, 8 mL) and ascorbic acid (0.1 m, 4 mL). Then, the reaction solution was kept at 60 °C for 4 h. After centrifugation (3500–7000 rpm, 10 min), the obtained precipitate was redispersed in CTAB solution (0.03–0.1 m, 20 mL) and left undisturbed at room temperature overnight. The supernatant was discarded, and the remaining precipitate was redispersed in water (10 mL). Subsequently, the CTAB solution (0.1 m, 0.2 mL), NH_3_·H_2_O (30 wt%, 0.8 mL), and H_2_O_2_ (0.1 m, 0.6 mL) were added, and the resultant solution was kept at room temperature overnight. The clear supernatant was then centrifuged for 10 min, and the precipitate was redispersed in deionized water (20 mL) for further use.

*Preparation of Au NBP/TiO_2_ Nanostructures*: The CTAB‐capped NBPs were first coated with poly(sodium 4‐styrenesulfonate) (PSS). Briefly, CTAB‐capped Au NBP solution (10 mL) was added dropwise to a PSS solution (molecular weight 70 000, 2 g L^−1^, 10 mL, 6 × 10^−3^ m NaCl), followed by stirring for 6 h. The excess PSS molecules were removed by centrifugation (5000–9000 rpm, 10 min), and the resultant pellet was redispersed in deionized water (0.2 mL). The NBP/TiO_2_ nanostructures were prepared as described in a previous study.^23^ TiCl_3_ solution (15 wt%, 0.2 mL, containing 20–30 wt% HCl) was added to deionized water (6 mL) under stirring, followed by the dropwise addition of NaHCO_3_ (0.93 m, 1.35 mL) and immediate addition of the concentrated NBP/PSS solution above (0.2 mL) to the mixture. After 30 min of stirring, the produced NBP/TiO_2_ nanostructures were precipitated by centrifugation (3000–5000 rpm, 10 min) and redispersed in deionized water (10 mL).

*Au NBP Nanostructure Characterization*: The extinction spectra of the Au NBP samples were measured using a Hitachi U‐3501 UV/visible/NIR spectrophotometer. The size and shape of the synthesized Au NBP samples were characterized using an FEI Tecnai Spirit microscope operated at 120 kV. The concentration of the Au NBP samples was determined with an Agilent ICP‐MS 7500a system (Tokyo, Japan).

*Cell Viability Assay*: Five thousand U‐87 MG cells were seeded into each well of a 96‐well plate. After 12 h of incubation, the medium in the wells was replaced with fresh medium containing drugs or NBP/TiO_2_ nanostructures. After further incubation for 48 or 72 h, the MTT assay or calcein AM staining was performed. For the MTT assay, the medium was discarded, and a fresh medium (100 µL) containing MTT (0.5 mg mL^−1^) was added. After 3 h of incubation, the medium was removed, and the purple formazan crystals were dissolved with dimethyl sulfoxide (DMSO) (150 µL). The absorbance at 540 nm was measured using a SpectraMax Paradigm multimode microplate reader (Molecular Devices, Sunnyvale, CA, USA). The cell viability of each sample relative to the control was calculated. For calcein AM staining, the medium in the wells was replaced with serum‐free α‐MEM containing calcein AM (1 × 10^−6^ m). After incubation for 30 min, the cells were washed with fresh medium, and an Olympus IX71 microscope was used to capture the images.

*Western Blotting*: After treatment, cells were washed with ice‐cold phosphate buffered saline (PBS) and lysed with the radioimmunoprecipitation assay (RIPA) lysis buffer containing the protease and phosphatase inhibitor cocktail at 4 °C for 15 min. The samples were centrifuged (12 000 rpm, 15 min) at 4 °C, and the supernatants were collected as the cell lysates. The protein concentration was determined by a Bradford protein assay. Equal amounts of protein (30 µg) from each sample were separated by sodium dodecyl sulfate‐polyacrylamide gel electrophoresis on 8–12% gels and transferred to polyvinylidene difluoride (PVDF) membranes. After incubation with primary antibodies followed by horseradish peroxidase‐conjugated secondary antibodies, the protein bands were developed and visualized by enhanced chemiluminescence (ECL) reagents.

*Colocalization Analysis*: Twelve thousand U‐87 MG cells stably expressing GFP‐LC3 were seeded into a confocal dish. After 12 h of incubation, the cells were treated with Rap (1 × 10^−6^ m) or NBP/TiO_2_ nanostructures (60 µg Au mL^−1^) for 24 h or with CQ (50 × 10^−6^ m) for 4 h, followed by LAMP1 immunostaining or live cell staining with LysoTracker Red. For LAMP1 immunostaining, the cells were fixed in 4% paraformaldehyde for 15 min followed by permeabilization with 0.1% Triton X‐100 in PBS for 10 min. The fixed preparations were blocked with 3% bovine serum albumin (BSA) in PBS for 1 h, then incubated with the primary antibody against LAMP1 (1:200) in 3% BSA for 1 h. The cells were then washed and incubated with Alexa Fluor 555‐conjugated secondary antibody (1:300) for 1 h. For LysoTracker red live cell imaging, the cells were stained with LysoTracker Red DND‐99 (75 × 10^−9^ m) in serum‐free α‐MEM for 20 min, then washed with fresh medium. For intracellular F‐actin distribution imaging, after the cells were fixed and permeabilized, they were stained with rhodamine phalloidin (1:500) for 30 min followed by Hoechst 33342 (100 ng mL^−1^) for 15 min. Images were captured by a Leica SP8 confocal microscope (Leica Microsystems, Wetzlar, Germany).

*DQ‐Red BSA Staining*: Cells seeded into a confocal dish were preincubated with DQ‐Red BSA (10 µg mL^−1^) for 12 h, then washed with PBS followed by incubation with BafA1 (10 × 10^−9^ m), Rap (1 × 10^−6^ m), NBP/TiO_2_ nanostructures (60 µg Au mL^−1^) or a combination of Rap and NBP/TiO_2_ nanostructures for 24 h. After extensive rinsing with PBS, the cells were observed under a confocal microscope.

*Cathepsin Activity Assay*: The cathepsin activities were determined as described in a previous study.^57^ Briefly, U‐87 MG cells were treated with NBP/TiO_2_ nanostructures (60 µg Au mL^−1^), Bor (10 × 10^−9^ m), a combination of Bor and NBP/TiO_2_ nanostructures, CA‐074 Me (10 × 10^−6^ m), E‐64‐D (20 × 10^−6^ m), Rap (1 × 10^−6^ m), or a combination of Rap and NBP/TiO_2_ nanostructures for 24 h, and then the cells were collected and lysed in lysis buffer (25 × 10^−3^ m 2‐(*N*‐morpholino)ethanesulfonic acid (MES), 1 × 10^−3^ m ethylenediaminetetraacetic acid (EDTA), 1 × 10^−3^ m dithiothreitol, 0.5% NP‐40, pH 5.0). Fifteen micrograms of total cellular protein was added to 100 µL of assay buffer (25 × 10^−3^ m MES, 1 × 10^−3^ m EDTA, 1 × 10^−3^ m dithiothreitol, pH 5.0) containing each substrate (Z‐RR‐AMC for CTSB, Bz‐Arg‐Gly‐Phe‐Phe‐Pro‐4M_2_NA for CTSD, Z‐FR‐AFC for CTSL, or Z‐GPR‐AMC for CTSK, 25 × 10^−6^ m). After 30 min of incubation at 37 °C, the fluorescence intensity of the solution with excitation at 380 nm and emission at 460 nm was detected by the plate reader. The cathepsin activity for each sample relative to the control was calculated. The direct effect of the NBP/TiO_2_ nanostructures on purified CTSB was also evaluated. Purified human CTSB (0.1 µg) was preincubated with NBP/TiO_2_ nanostructures (60 or 120 µg Au mL^−1^) in 100 µL of assay buffer at 37 °C for 12 h, followed by incubation with Z‐RR‐AMC (25 × 10^−6^ m) at 37 °C for 30 min. After centrifugation (12 000 rpm, 10 min), the fluorescence intensity of the supernatant was detected, and the relative CTSB activity was calculated as described above.

*Proteasome Activity Assay*: The proteasome activity was determined as described previously.^58^ Cell were lysed in lysis buffer (50 × 10^−3^ m 4‐(2‐hydroxyethyl)piperazine‐1‐ethanesulfonic acid (HEPES), 10 × 10^−3^ m NaCl, 1.5 × 10^−3^ m KCl, 1 × 10^−3^ m EDTA, 1 × 10^−3^ m dithiothreitol, 1 × 10^−3^ m ATP, 250 × 10^−3^ m sucrose, 0.5% NP‐40, pH 7.4). Fifteen micrograms of crude protein extract was added to 100 µL of assay buffer (50 × 10^−3^ m HEPES, 10 × 10^−3^ m NaCl, 1.5 × 10^−3^ m KCl, 1 × 10^−3^ m EDTA, 1 × 10^−3^ m dithiothreitol (DTT), 1 × 10^−3^ m ATP, 250 × 10^−3^ m sucrose, pH 7.4) containing each substrate (Suc‐LLVY‐AMC for chymotrypsin‐like, Z‐LLE‐AMC for caspase‐like, or Boc‐LRR‐AMC for trypsin‐like activity of proteasome, 25 × 10^−6^ m). After 30 min of incubation at 37 °C, the fluorescence intensity with excitation at 380 nm and emission at 460 nm was detected by a plate reader. The proteasome activity of each sample relative to the control was calculated.

*Cotreatment with NBP/TiO_2_ Nanostructures with Bor or MG‐132*: Five thousand U‐87 MG cells were seeded into each well of a 96‐well plate and incubated for 12 h. The cells were then treated with Bor (0–40 × 10^−9^ m) or MG‐132 (0–2 × 10^−6^ m) in the presence or absence of NBP/TiO_2_ nanostructures (30 or 60 µg Au mL^−1^) for 48 h. The cell viability was determined by the MTT assay.

*Apoptosis Assay*: In vitro caspase‐3 activity was assayed in a 96‐well plate. For each group, 15 µg of total cellular protein was mixed with 89 µL of assay buffer (20 × 10^−3^ m HEPES, 2.5 × 10^−3^ m MgCl_2_, 10 × 10^−3^ m KCl, 1 × 10^−3^ m EDTA, 1 × 10^−3^ m EGTA, 1 × 10^−3^ m dithiothreitol, pH 7.4) plus caspase‐3 substrate (Ac‐DEVD‐AMC, 1 mg mL^−1^, 1 µL). The mixture was then incubated at 37 °C in darkness for 1 h. The fluorescent intensity was measured using a plate reader with excitation at 355 nm and emission at 460 nm. The relative caspase‐3 activity for each sample was presented relative to the control cells.

The intracellular Ca^2+^ level and morphology of mitochondria were observed through live cell staining with Fluo‐4 AM (1 × 10^−6^ m, 30 min) and MitoTracker Green FM (200 × 10^−9^ m, 30 min), respectively, in a 24‐well plate. Fluorescent images were captured.

*Combination of Photothermal Therapy and Bor Treatment*: Five thousand U‐87 MG cells were seeded into each well of a 96‐well plate. After 12 h of incubation, the culture medium was replaced with phenol red‐free α‐MEM (100 µL) containing NBP/TiO_2_ nanostructures (30 µg Au mL^−1^) and/or Bor (10 × 10^−9^ m) to prevent light absorption by phenol red, followed by further incubation for 24 h. For photothermal ablation, the covers of the plates were removed to avoid the reflection of laser light by the plastic cover. The designated wells were exposed to a continuous‐wave semiconductor diode laser (808 nm, MDL‐N‐808‐10 W, Changchun New Industries Optoelectronics Tech. Co., Ltd., China) for 2 min. The laser power density was 3.7 W cm^−2^, and the laser spot diameter was ≈6 mm, which is equal to that of the bottom of the well in the 96‐well plate. The cells were further incubated for 24 h. For comparison, cells not undergoing the photothermal therapy treatment were also subjected to Bor (10 × 10^−9^ m) for 48 h. The MTT assay and calcein AM staining were finally used to determine the cell viability.

*Statistical Analysis*: Results are expressed as the mean ± standard error of the mean (S.E.M.) of at least three independent experiments. The statistical significance of differences was evaluated by one‐way analysis of variance (ANOVA) followed by Tukey's post hoc test. A *P* value of <0.05 was considered statistically significant.

## Conflict of Interest

The authors declare no conflict of interest.

## Supporting information

Supplementary
