# Supplementary material for: Titania‐Coated Gold Nano‐Bipyramids for Blocking Autophagy Flux and Sensitizing Cancer Cells to Proteasome Inhibitor‐Induced Death
Source: Adv Sci (Weinh). 2017 Dec 1;5(3):1700585. doi: 10.1002/advs.201700585 (PMC5867123; doi:10.1002/advs.201700585)
Supplement: Supplementary file 1 — Supplementary [file ADVS-5-1700585-s001.pdf]

Copyright WILEY-VCH Verlag GmbH & Co. KGaA, 69469 Weinheim, Germany, 2017.

ADVANCED SCIENCE
Open Access

Supporting Information

for *Adv. Sci.,* DOI: 10.1002/advs.201700585

Titania-Coated Gold Nano-Bipyramids for Blocking Autophagy Flux and Sensitizing Cancer Cells to Proteasome Inhibitor-Induced Death

Hong-Ye Wan, Jian-Li Chen, Xingzhong Zhu, Liang Liu, Jianfang Wang, and Xiao-Ming Zhu**

Copyright WILEY-VCH Verlag GmbH & Co. KGaA, 69469 Weinheim, Germany, 2015.

Supporting Information

Titania-Coated Gold Nanobipyramids for Blocking Autophagy Flux and Sensitizing Cancer Cells to Proteasome Inhibitor-Induced Death

Hong-Ye Wan, Jian-Li Chen, Xingzhong Zhu, Liang Liu, Jianfang Wang, and Xiao-Ming Zhu**

H.-Y. Wan, J.-L. Chen, Prof. L. Liu, Prof. X.-M. Zhu
State Key laboratory of Quality Research in Chinese Medicine, Macau Institute for Applied Research in Medicine and Health
Macau University of Science and Technology
Avenida Wai Long, Taipa, Macau SAR, China
E-mail: xmzhu@must.edu.mo
Dr. X. Z. Zhu, Prof. J. F. Wang
Department of Physics
The Chinese University of Hong Kong
Shatin, Hong Kong SAR, China
E-mail: jfwang@phy.cuhk.edu.hk

I. SUPPORTING MATERIALS AND METHODS

Materials: LysoTracker Red DND-99, MitoTracker Green FM, calcein AM, Fluo-4 AM, Hoechst 33342, rhodamine phalloidin, DQ-Red BSA, Alexa Fluor 555-conjugated goat anti-rabbit second antibody, geneticin, Trizol reagent and alpha-modified Minimum Essential Medium (α-MEM) were purchased from Thermo Fisher Scientific (Walthan, MA, USA). Hilymax was purchased form Dojindo Laboratories (Kumamoto, Japan). Bafilomycin A1 (BafA1) was purchased from LC laboratories (Woburn, MA, USA). Rapamycin (Rap), MG-132, and bortezomib (Bor) were obtained from Selleck Chemicals (Houston, TX, USA). Chloroquine diphosphate (CQ) was purchased from TCI (Shanghai, China). CellTiter-Glo luminescent cell viability assay kit was purchased from Promega Corporation (Madison, WI, USA). Bz-Arg-Gly-Phe-Phe-Pro-4M$_2$NA was purchased from Sigma-Aldrich (St. Louis, MO, USA). Human purified cathepsin B (CTSB), Ac-DEVD-AMC, Z-RR-AMC, Z-GPR-AMC, Z-FR-AFC, Z-LLE-AMC, Suc-LLVY-AMC, Boc-LRR-AMC, CA-074 Me, and E-64-D were

purchased from Enzo Life Sciences (Farmingdale, NY, USA). RIPA lysis buffer, antibodies (anti-LC3 (2775), anti-p62 (5114), anti-LAMP1 (9091), anti-mTOR (2972), anti-p-mTOR (2971), anti-AMPK (2532), anti-p-AMPK (2531), anti-Akt (9272), anti-p-Akt (4060), anti-p70S6K (9202), anti-p-p70S6K (9205), anti-cathepsin B (31718), and anti-BiP (3177)) were purchased from Cell Signaling Technology (Beverly, MA, USA). Antibodies (anti-ubiquitin (sc-8017), anti-β-actin (sc-47778), goat anti-rabbit IgG-HRP (sc-2004), and goat anti-mouse IgG-HRP (sc-2005)) were purchased from Santa Cruz (Dallas, Texas, TX, USA). Human glioblastoma U-87 MG cells were obtained from American Type Culture Collection (ATCC, Manassas, VA, USA).

Preparation of NBP/mSiO$_2$ Nanostructures: CTAB (0.1 M, 0.07 mL) and NaOH (0.1 M, 0.12 mL) were added to the CTAB-capped Au NBP solution (10 mL). After the solution was mixed thoroughly, three aliquots of tetraethyl orthosilicate (TEOS, 10 v/v% in ethanol, 20 μL) were added at a 2 h interval while the mixture solution was kept under shaking at 45 °C in a thermostat steam bath vibrator. The NBP/mSiO$_2$ nanostructures were obtained by centrifugation (7000 rpm, 10 min), then washed first with methanol containing HCl (1 M) and then with deionized water to remove the excess CTAB molecules, and finally redispersed in deionized water (10 mL).

Preparation of NBP/PEG Nanostructures: Methoxy-PEG-thiol (mPEG-SH, molecular weight 5000, 1×10^{-3} M, 0.5 mL, RAPP polymere, German) was mixed with CTAB-capped Au NBP solution (10 mL). The resultant solution was kept under vigorous stirring for at least 6 h. The excess mPEG-SH molecules were removed by centrifugation (7000 rpm, 10 min). The obtained NBP/PEG nanostructures were redispersed in deionized water (10 mL).

Preparation of NBP/dSiO$_2$ Nanostructures: The NBP/PEG nanostructure solution (10 mL) obtained above was centrifuged at 7000 rpm for 10 min. The resultant pellet was redispersed in ethanol (7.5 mL), followed by the addition of deionized water (2.25 mL) and $NH_3 \cdot H_2O$ (30%, 0.15 mL). Three aliquots of TEOS (10 v/v% in ethanol, 20 μL) were injected

at a 1 h interval under continuous ultrasonication. The NBP/dSiO_2 nanostructures were obtained by centrifugation (7000 rpm, 10 min) and redispersed in deionized water (10 mL).

Cellular Uptake Observation: Eight thousand cells were seeded into each well of a 24-well plate. After 12 h of incubation, the culture medium was replaced with fresh medium containing the NBP samples (60 μg Au mL^{-1}, 0.5 mL). After the cells were further incubated for 24 h, they were then washed with PBS extensively to remove any nanoparticles adsorbed on the cell surface. The cells were subsequently observed under an Olympus IX71 microscope (Melville, NY, USA) under bright field.

Intracellular Au Content Assay: U-87 MG cells were seeded into each well of a 6-well plate. When the cells reached 80% confluence, the culture medium was replaced with fresh medium containing the NBP/TiO_2 nanostructures (30 μg Au mL^{-1}, 2 mL) with three different sizes. After 24 h of incubation, cells were washed, collected and counted. The cells were subsequently centrifuged at 4500 rpm for 10 min. The obtained cell pellets were redispersed in *aqua regia* (200 μL) to digest the nanoparticles. The digested solution was diluted with deionized water. The Au concentration of the resultant solution was determined with Agilent ICP-MS 7500a system (Tokyo, Japan). The Au amount per cell was calculated.

Intracellular ATP Assay: Cells were lysed with Triton X-100 (1%, 100 μL) and centrifuged at 12,000 rpm for 10 min. 70 μL supernatant of each sample was transferred to a 96-well white plate. CellTiter-Glo reagent (70 μL) was then added, and after mixed thoroughly, the mixture was incubated for 10 min followed by luminescence measurement using the SpectraMax Paradigm multi-mode microplate reader.

Quantitative Real-Time PCR Analysis: Total mRNA of each group was extracted using the Trizol reagent. Reverse transcription PCR was done using Transcriptor First-Strand cDNA Synthesis kit (Roche, Mannheim, Germany). Real-time PCR analysis was performed in a ViiA 7 real-time PCR system (Thermo Fisher Scientific, Walthan, MA, USA) using FastStart Universal SYBR Green Mater (Roche). β-actin was amplified as normalizer and fold

change in expression of pro-CTSB mRNA relative to β-actin was calculated according to $2^{-\Delta\Delta ct}$ method. The primers for real-time PCR were as followings:

pro-CTSB (human): 5′-CTGTCGGATGAGCTGGTCAAC-3′ (forward) and 5′-TCGGTAAACATAACTCTCTGG GG-3′ (reverse).

β-actin (human): 5’-TCACCCACACTGTGCCCATCT-3’(forward) and 5’-GTGAGG ATCTTCATGAGGTAGTCAGTC-3’ (reverse).

CTSB Binding Assay: Total cell lysate was prepared by lysing the cells with lysis buffer. Each NBP sample (20 μg Au) was added to the cell lysate solution (2.7 μg μL^{-1}, 70 μL), and the mixture was incubated at 4 °C for 12 h. After centrifugation (12,000 rpm, 15 min), the supernatant was collected. Western blotting was used to detect the CTSB level in the supernatant for each sample.

II. SUPPORTING TABLE

Table S1. Sizes, and TiO_2 Coating Thicknesses of Three NBP/TiO_2 Nanostructures.

	NBP length (nm)	NBP diameter (nm)	Aspect ratio	TiO_2 thickness (nm)
NBP1/TiO_2	47 ± 4	20 ± 2	2.3 ± 0.2	14 ± 2
NBP2/TiO_2	95 ± 5	33 ± 2	2.9 ± 0.2	15 ± 2
NBP3/TiO_2	142 ± 8	42 ± 3	3.4 ± 0.2	14 ± 2

III. SUPPORTING FIGURES

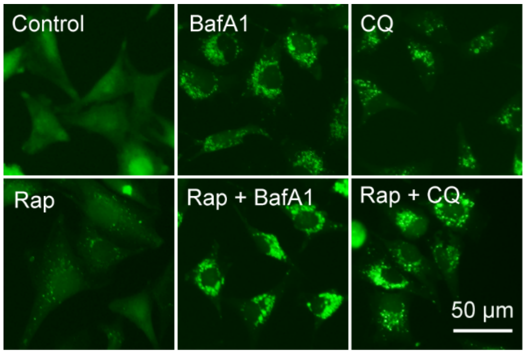

Figure S1. Co-treatment with autophagy inducer Rap and autophagy inhibitor BafA1 or CQ significantly induce autophagosome accumulation. GFP-LC3-expressing U-87 MG cells were

treated with Rap (1×10^{-6} M) in the presence or absence of BafA1 (10×10^{-9} M) or CQ (20×10^{-6} M) for 24 h, followed by fluorescent imaging.

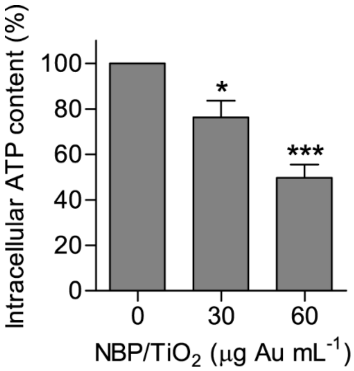

Figure S2. NBP/TiO_2 nanostructures decrease intracellular ATP content. U-87 MG cells were incubated with NBP/TiO_2 nanostructures for 48 h, the intracellular ATP was analyzed with a CellTiter-Glo luminescent ATP assay. The data shown represent the mean ± S.E.M., * $P < 0.05$, and *** $P < 0.001$.

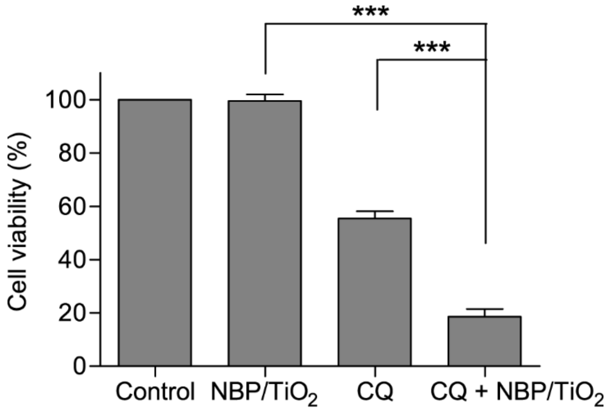

Figure S3. NBP/TiO_2 nanostructures synergistically enhance the cytotoxic effect of CQ. U-87 MG cells were incubated with CQ (50×10^{-6} M) and/or NBP/TiO_2 nanostructures (30 μg Au mL^{-1}) for 48 h, and the cell viability was assessed by the MTT assay. The shown data represent the mean ± S.E.M., *** $P < 0.001$.

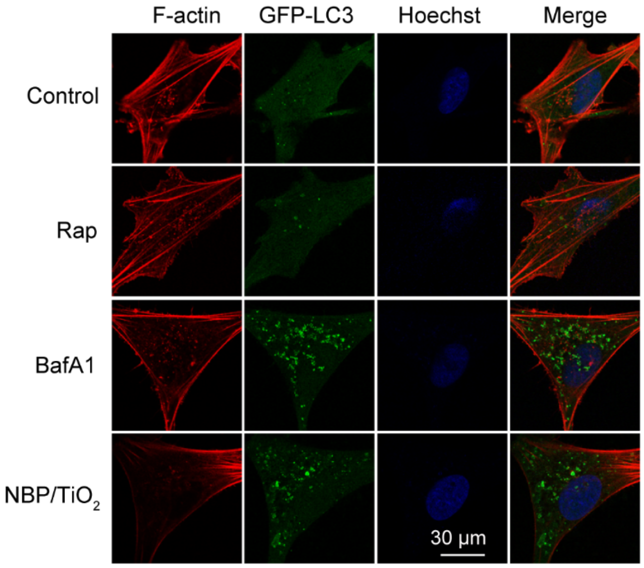

Figure S4. NBP/TiO_2 nanostructures disrupt F-actin distribution. U-87 MG cells stably expressing GFP-LC3 were treated with NBP/TiO_2 nanostructures (60 μg Au mL^{-1}), Rap (1×10^{-6} M), and BafA1 (10×10^{-9} M) for 24 h. The cells were then stained with phalloidine and the intracellular F-actin distribution was observed under a confocal microscope.

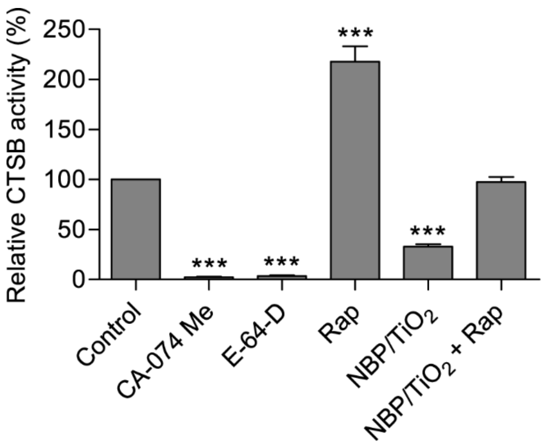

Figure S5. NBP/TiO_2 nanostructures inhibit the CTSB activity stimulated by Rap. U-87 MG cells were treated with CA-074 Me (10×10^{-6} M), E-64-D (20×10^{-6} M), NBP/TiO_2 nanostructures (60 μg Au mL^{-1}) and/or Rap (1×10^{-6} M) for 24 h, and CTSB activity was determined.

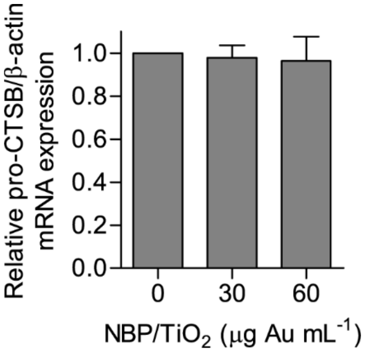

Figure S6. NBP/TiO_2 nanostructures don't affect the mRNA expression of pro-CTSB. Relative pro-CTSB mRNA level (compared with β-actin) was analyzed by quantitative real time-PCR.

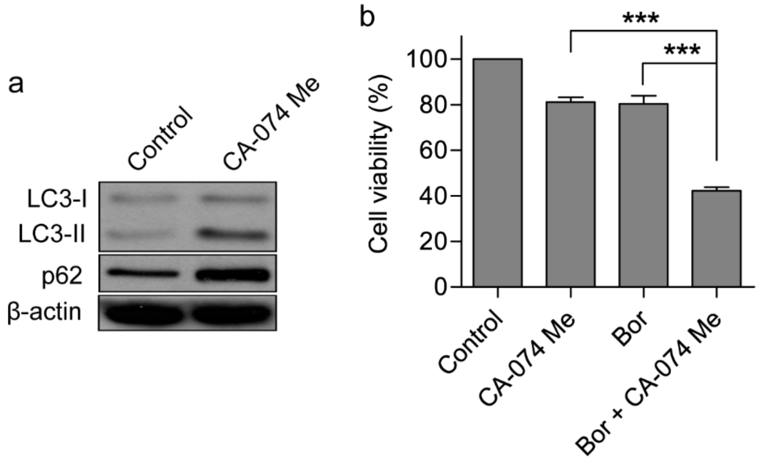

Figure S7. Autophagy inhibition by CA-074 Me sensitizes cells to Bor-induced cytotoxicity. (a) Autophagic flux inhibition by CA-074 Me. Western blotting analysis of LC3 and p62 expression of U-87 MG cells after incubation with CA-074 Me (10×10^{-6} M). (b) CA-074 Me synergistically enhances Bor's cytotoxicity. U-87 MG cells were treated with Bor (10×10^{-9} M) and/or CA-074 Me (10×10^{-6} M) for 48 h, and the MTT assay was performed to evaluate the cell viability. The data shown represent the mean ± S.E.M., *** $P < 0.001$.
